# Giant vulvar neurofibroma in a 19-year-old female: A rare peripheral nerve sheath tumor

**DOI:** 10.1016/j.radcr.2026.04.024

**Published:** 2026-05-07

**Authors:** Muffrih Mohamed Haresi

**Affiliations:** Department of Radiology, King Fahd Central Hospital, Jizan, Saudi Arabia

**Keywords:** Vulvar neurofibroma, Peripheral nerve sheath tumor, MRI, Vulvar mass, S100 immunoexpression, Case report

## Abstract

Neurofibromas are benign peripheral nerve sheath tumors that infrequently involve the vulva. Giant vulvar neurofibromas are exceedingly rare, particularly in young patients, and may pose diagnostic challenges due to their unusual location and broad differential diagnosis. We report the case of a 19-year-old female who presented with a slowly enlarging left vulvar mass. Magnetic resonance imaging (MRI) demonstrated a large, well-circumscribed, lobulated soft-tissue lesion with signal characteristics suggestive of a benign peripheral nerve sheath tumor, without invasion of adjacent structures. Complete surgical excision was performed, and histopathological examination confirmed the diagnosis of neurofibroma using S100 immunohistochemical staining. This case highlights the pivotal role of MRI in lesion characterization, surgical planning, and differentiation from other vulvar masses.

## Introduction

Neurofibromas are benign tumors arising from peripheral nerves and are commonly associated with neurofibromatosis type I; however, isolated sporadic lesions also occur [1]. Involvement of the vulva is extremely uncommon, with only a limited number of cases reported in the literature [2]. Giant vulvar neurofibromas are particularly rare and may present diagnostic uncertainty due to overlap in clinical and imaging features with other benign and malignant vulvar lesions [3]. Imaging, especially MRI, plays a crucial role in lesion characterization, assessment of local extent, and preoperative planning [4].

## Case presentation

A 19-year-old female presented with a progressively enlarging left vulvar mass that had been increasing in size over several years. The patient reported no pain, ulceration, or discharge. There was no known personal or family history of neurofibromatosis. On physical examination, a large, pendulous, non-tender mass arising from the left vulva was noted, with intact overlying skin.

## Imaging findings

Pelvic MRI revealed a large, well-defined, lobulated soft-tissue mass arising from the left vulvar region. The lesion appeared hypointense on T1-weighted images and heterogeneously hyperintense on T2-weighted images. No areas of hemorrhage, necrosis, or fat signal were identified. The mass demonstrated a superficial origin, with displacement rather than invasion of adjacent soft tissues. There was no extension into the pelvic cavity or involvement of pelvic organs. The imaging features were suggestive of a benign peripheral nerve sheath tumor.

**Fig. 1:**
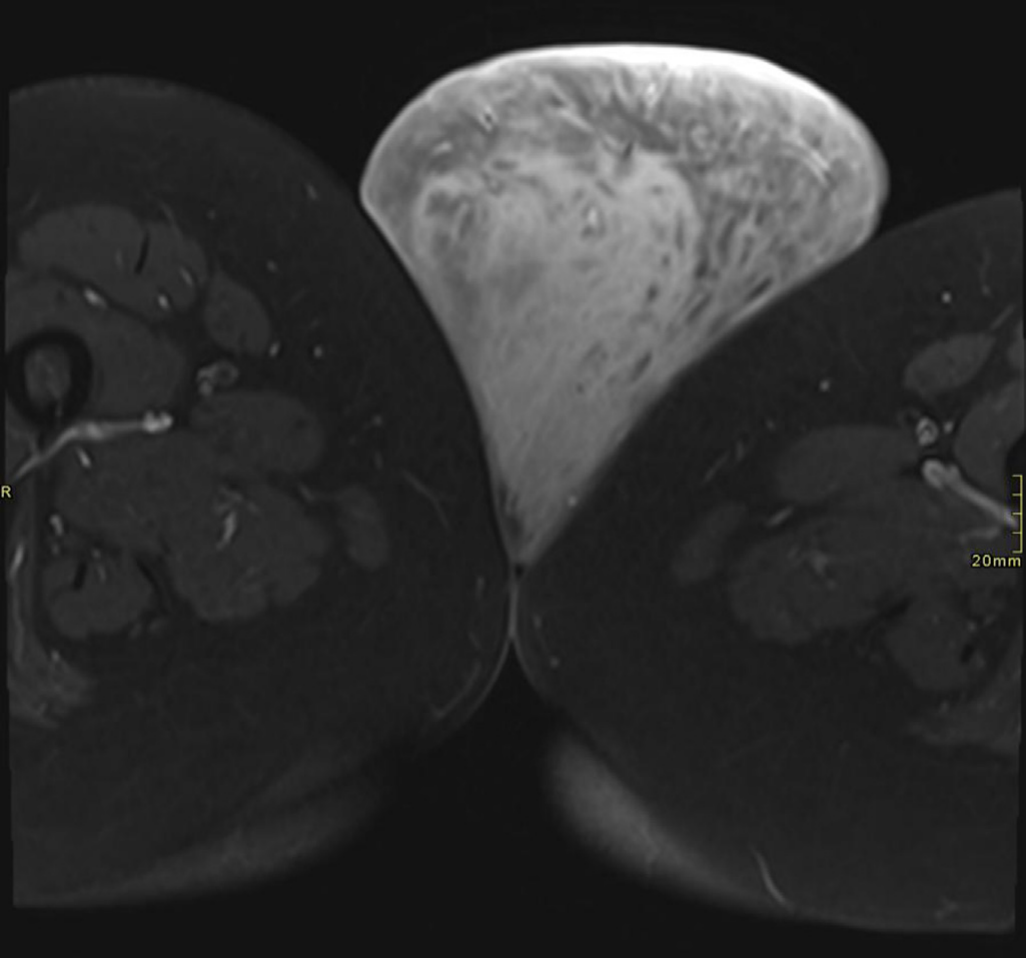
Axial T2-weighted MRI showing a large, well-defined, heterogeneously hyperintense mass arising from the left vulvar region, displacing adjacent soft tissues without invasion.

**Fig. 2:**
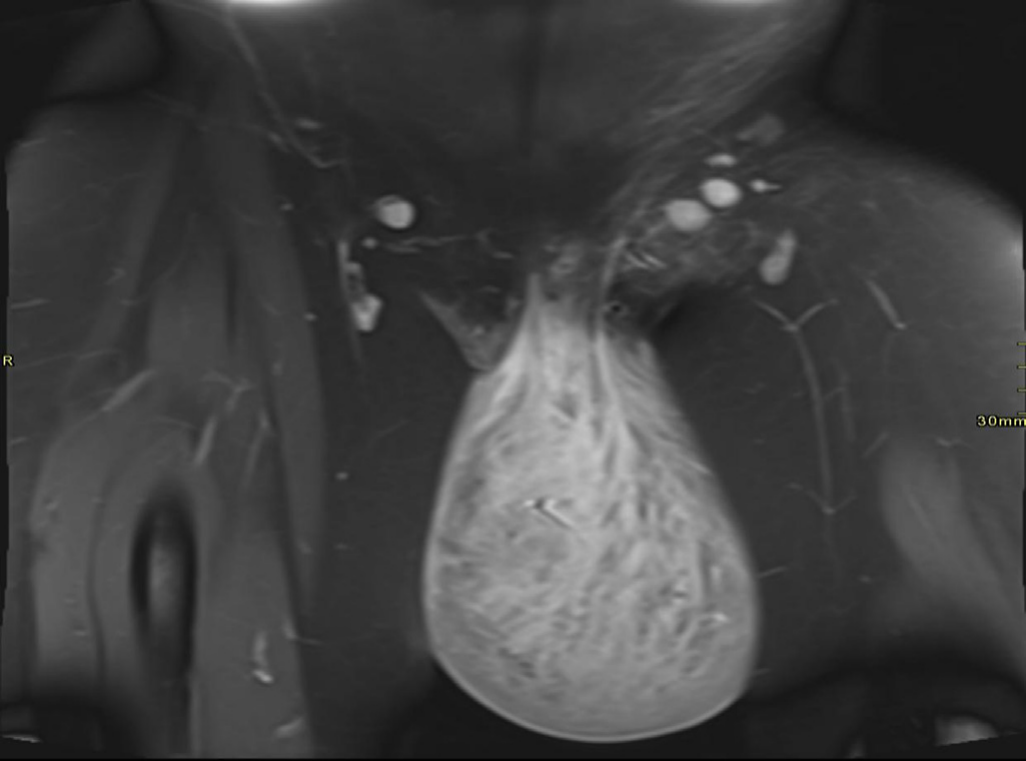
Coronal T2-weighted MRI demonstrates the pendulous configuration of the vulvar mass extending inferiorly from the left vulva.

As shown in Figure 1, axial T2-weighted MRI demonstrates a large, well-defined heterogeneously hyperintense left vulvar mass displacing adjacent soft tissues without invasion. Figure 2 illustrates the coronal view, highlighting the pendulous inferior extension of the lesion from the left vulva.

**Fig. 3:**
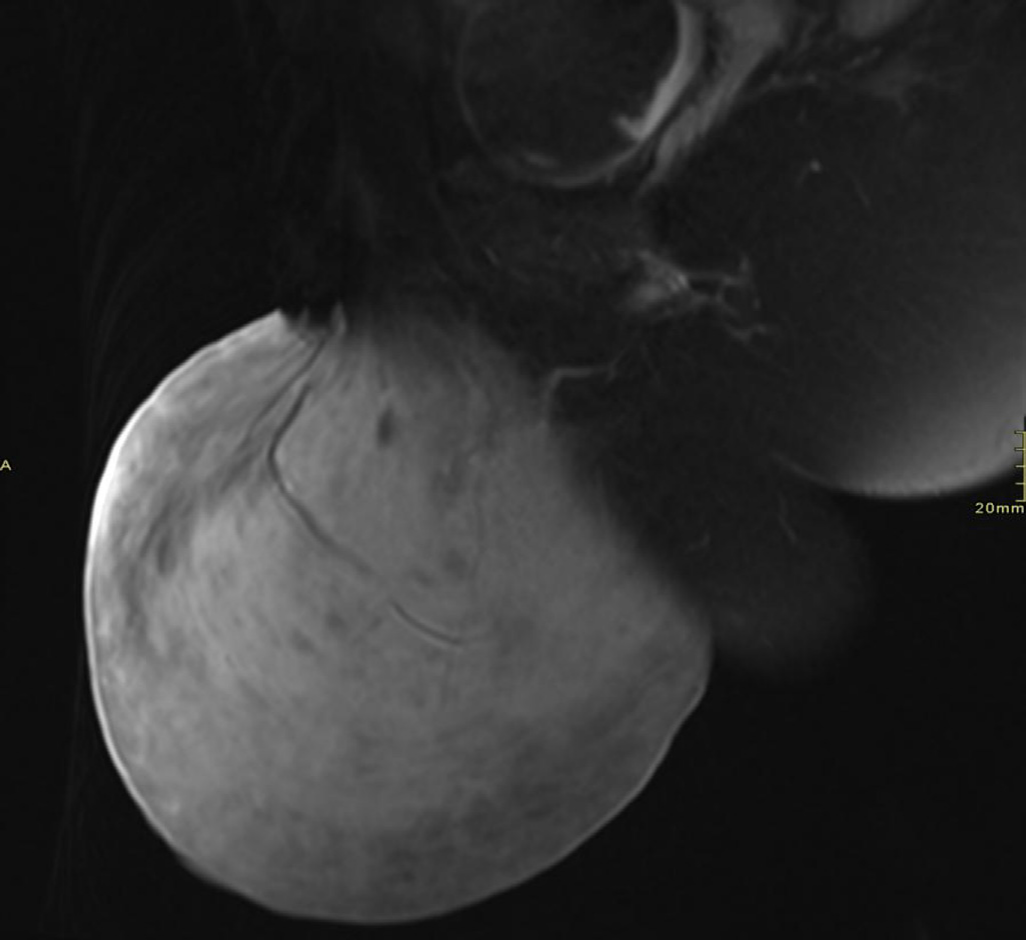
Sagittal post contrast MRI image illustrates the superficial origin of the lesion from the vulvar soft tissues with heterogonous post contrast enhancement.

Post-contrast sagittal MRI in Figure 3 demonstrates the superficial origin of the lesion with heterogeneous enhancement.

## Management

**Fig. 4:**
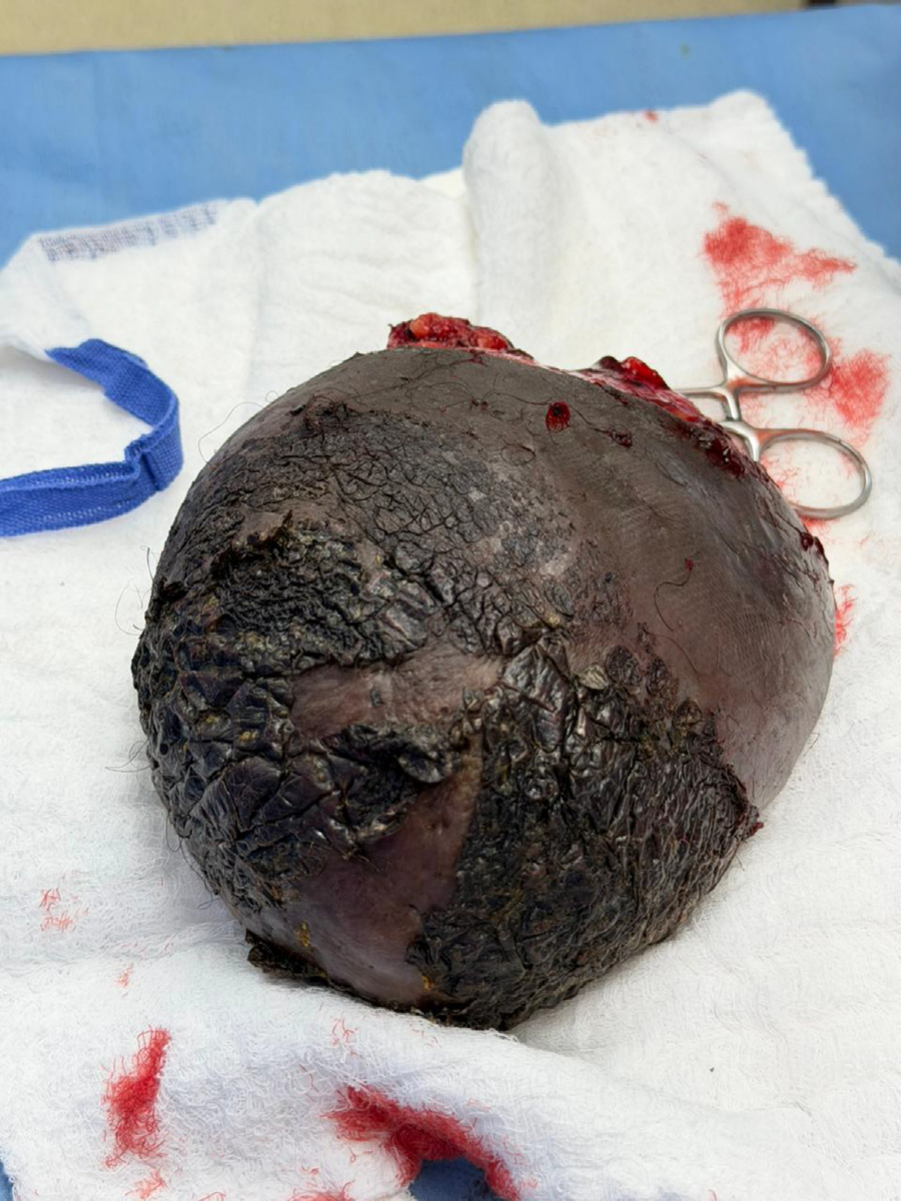
Gross specimen photograph shows a large, well-circumscribed excised left vulvar mass with darkened overlying skin and intact surface.

The patient underwent complete surgical excision of the mass with preservation of surrounding vulvar structures. The postoperative course was uneventful. The gross appearance of the excised specimen is shown in Figure 4.

## Histopathological findings

Excision biopsy of the lesion was done and sent to pathology department for histopathological examination. On gross examination one grey white soft tissue piece, measuring 15×10.5×7.5 cm was noted covered by skin with surface crustation. Light microscopic examination revealed normal appearing epidermis with surface crustation, underlying subepithelium shows non-encapsulated tumor composed of a mixed proliferation of spindle shaped cells with wavy nuclei and bland nuclear chromatin in a edematous stroma. No atypia or necrosis was seen in the sections examined.

A diagnosis of neurofibroma was made on microscopic examination which was confirmed on immunohistochemistry. On immunohistochemistry strong positivity for S-100 was noted. while CD34 showed faint focal expression.

**Fig. 5:**
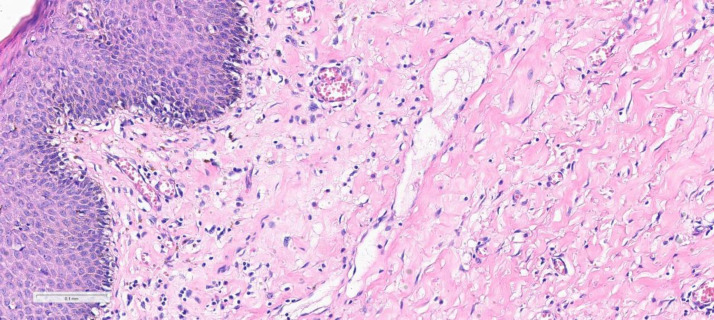
Showing epidermis with underlying neurofibroma spindle cells and charactristic wavy nuclei surrounding blood vessles in an edematous stroma . (H&E, x200).

Histopathological examination in Figure 5 demonstrates spindle cells with characteristic wavy nuclei within an edematous stroma.

**Fig. 6:**
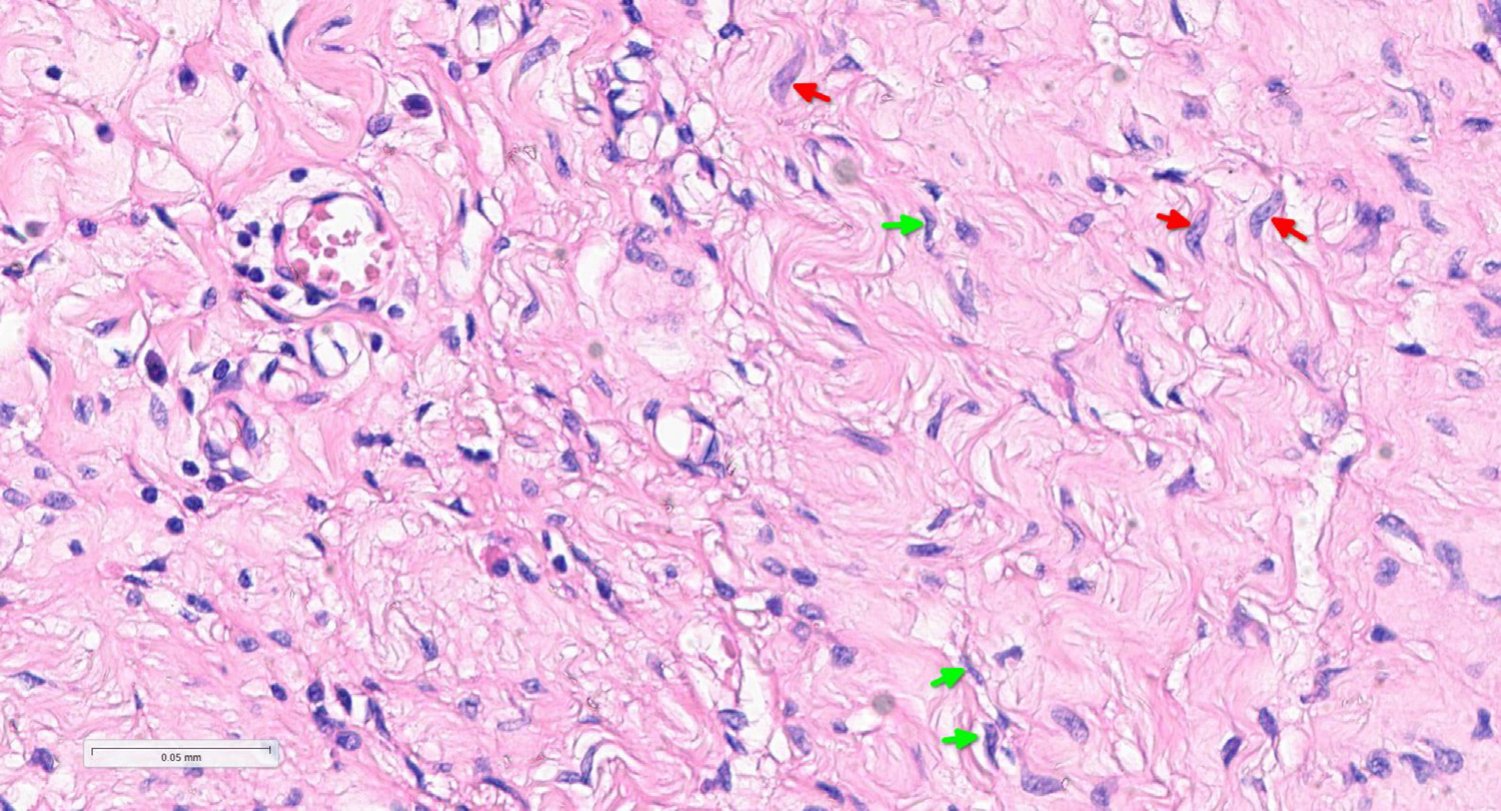
Showing neurofibroma with characteristic spindle cells (red arrows) and wavy nuclei (green arrows). (H&E, x400).

Higher magnification in Figure 6 further highlights the spindle cell morphology and wavy nuclei typical of neurofibroma.

**Fig. 7:**
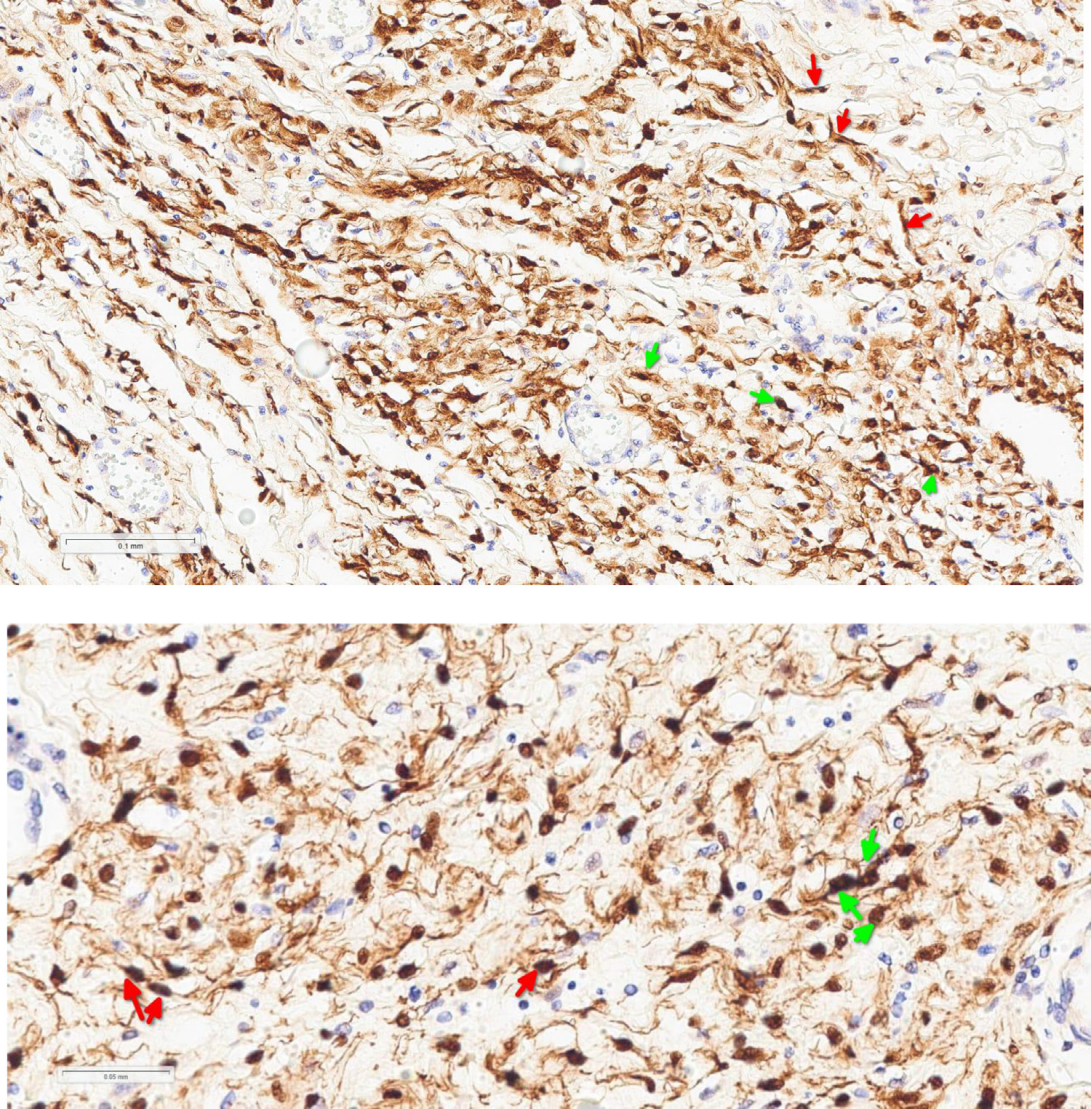
A (top, x200) and B (bottom, x400): Showing spindle cells of neurofibroma (red arrows) with diffuse, strong nuclear & membranous immunoexpression of S100 (green arrows) (S100 immunoexpression, Mayer's H,).

Immunohistochemical staining in Figure 7A and B shows diffuse strong S100 positivity, supporting the diagnosis of neurofibroma.

**Fig. 8:**
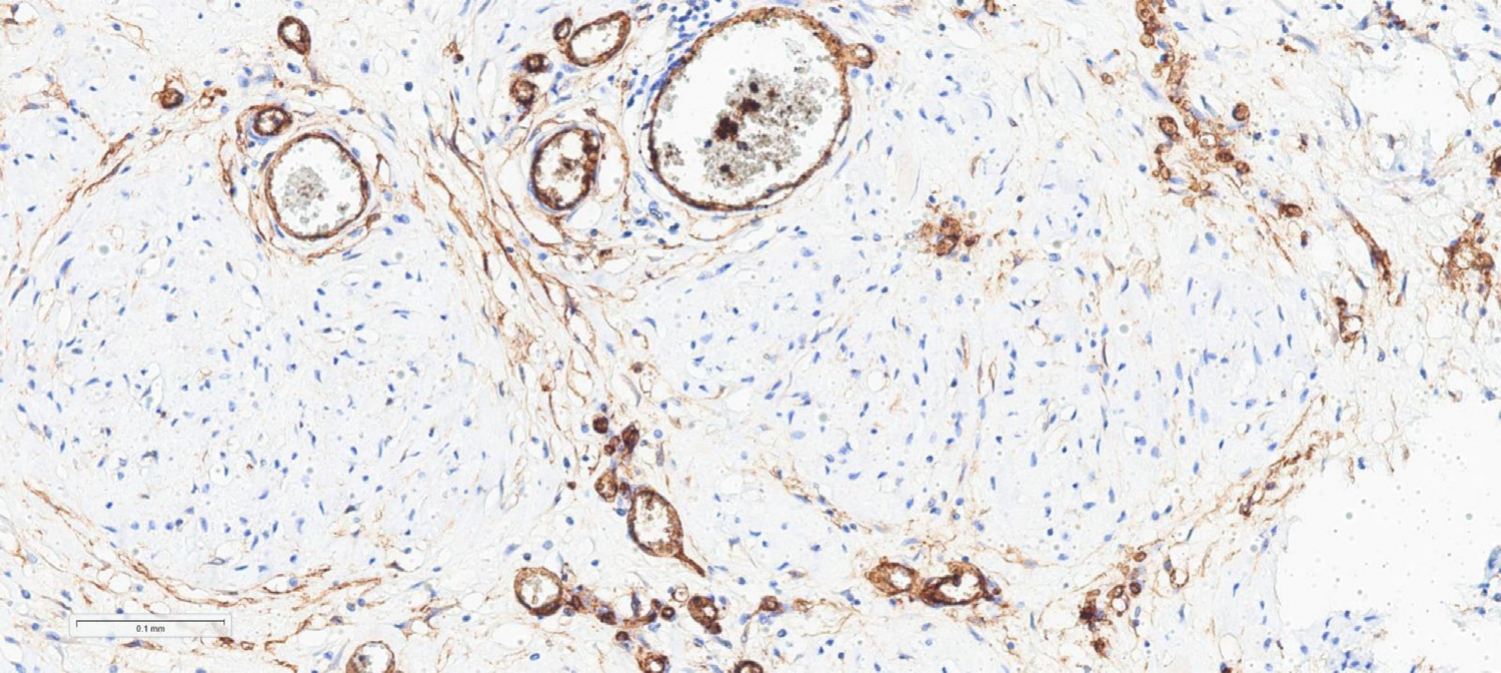
Showing spindle cells of neurofibroma with faint focal membranous immunoexpression expression of CD34 (CD34 immunoexpression, Mayer's H. x200).

Figure 8 demonstrates faint focal CD34 immunoexpression in tumor cells.

## Discussion

Vulvar neurofibromas are rare benign tumors that may present as slow-growing, painless masses [5]. When large, they can cause functional or cosmetic concerns [6]. The differential diagnosis of vulvar soft-tissue masses includes Bartholin gland cysts, lipomas, aggressive angiomyxomas, leiomyomas, and soft-tissue sarcomas [7]. MRI is the imaging modality of choice for evaluating vulvar lesions due to its superior soft tissue contrast and ability to delineate lesion extent and tissue characteristics [8].

Typical MRI features of neurofibromas include low signal intensity on T1-weighted images and high signal intensity on T2-weighted images, often with a well-defined margin and absence of invasive features [9]. Recognition of these imaging characteristics can guide appropriate surgical management and avoid overly aggressive treatment [10].

Complete surgical excision is curative in most cases, with an excellent prognosis and low recurrence rate [11].

## Conclusion

Although extremely rare, vulvar neurofibroma should be considered in the differential diagnosis of vulvar masses, especially in young patients presenting with slow-growing, painless lesions. MRI plays a critical role in accurate diagnosis, assessment of lesion extent, and surgical planning. Histopathological and immunohistochemical evaluation remains essential for definitive diagnosis.

## Patient consent

Written informed consent was obtained from the patient for publication of this case report.
